# Hsa_circ_0008259 modulates miR-21-5p and PDCD4 expression to restrain osteosarcoma progression

**DOI:** 10.18632/aging.203769

**Published:** 2021-12-14

**Authors:** Kai Guan, Shizhang Liu, Keke Duan, Xiaoxia Zhang, Huitong Liu, Bingqiang Xu, Xi Wang, Xin Jin

**Affiliations:** 1 The Second Department of Orthopaedics, The First People's Hospital of Xianyang, Xianyang 712000, Shaanxi Province, China; 2 Department of Orthopedics, Shaanxi Provincial People’s Hospital, The Third Affiliated Hospital of Xi’an Jiaotong University, Xi’an 710068, Shaanxi Province, China

**Keywords:** circ_0008259, miR-21-5p, PDCD4, osteosarcoma

## Abstract

Background: Osteosarcoma (OS) is one of the most common primary bone tumors in children and adolescents. However, the molecular mechanism of OS tumorigenesis is still little known. Circular RNA (circRNA) is a key player in the progression of many cancers. This study is performed to decipher the role and mechanism of circ_0008259 in the progression of OS.

Methods: A differentially expressed circRNA, circ_0008259, was screened out by analyzing the expression profile of circRNA in OS tissue. Circ_0008259, miR-21-5p and programmable cell death 4 (PDCD4) mRNA expression levels in OS tissues and cells were detected by qRT-PCR. Cell viability, metastatic potential and apoptosis were evaluated by cell counting kit-8 assay, Transwell and flow cytometry. The targeting relationship between circ_0008259 and miR-21-5p, and miR-21-5p and PDCD4 mRNA was analyzed and probed by bioinformatics analysis and dual-luciferase reporter assay, RNA-binding protein immunoprecipitation assay and RNA-pull down assay. The regulatory effects of circ_0008259 and miR-21-5p on PDCD4 protein expression in OS cells were detected by Western blot assay.

Results: Circ_0008259 expression and PDCD4 expression were down-regulated and miR-21-5p expression was elevated in the OS tissues and cells. Functional experiments showed that circ_0008259 overexpression significantly inhibited the proliferation and metastatic potential of OS cells and promoted the apoptosis. Besides, PDCD4 was validated as the target gene of miR-21-5p, and circ_0008259 could competitively bind to miR-21-5p, thus up-regulating PDCD4 expression in OS cells.

Conclusions: Circ_0008259 suppresses OS progression via regulating miR-21-5p/PDCD4 axis.

## INTRODUCTION

Osteosarcoma (OS) mainly occurs in adolescents, often leading to disability or death [1–3]. The prognosis of OS is very poor ensuing from metastasis and drug resistance [4]. Therefore, it is crucial to look for new therapy targets to intensify the efficacy of OS treatment and improve the prognosis of OS.

Circular RNA (circRNA) is the non-coding RNA generated by back-splicing that forms a covalently closed loop without 3′ and 5′ ends [5]. In recent years, many dysregulated circRNAs in the pathological process of many diseases have been identified [6, 7]. The specific expression and biological function of circRNAs in tumors enable them with potentials as biomarkers and therapeutic targets. For example, circ_0005556 expression is markedly declined in gastric cancer, which is associated with tumor differentiation status, TNM stage, lymph node metastasis, and overall survival time of sufferers [8]. Certain circRNA, as reported, is abnormally expressed in OS tissues. For example, circ-ITCH is silenced in OS as a tumor suppressor, while up-regulated circ-0003998as a tumor promoter [9, 10]. How circ_0008259 works in OS is undefined, nevertheless.

MicroRNAs (miRNAs), conceptually a category of endogenous RNA with around 20-22*nt*, are modulators of many physiological and pathological processes. CircRNA can sponge miRNA to monitor the targets of miRNA post-transcriptionally [11]. For instance, circ_001621 accelerates OS cell proliferation and migration via adsorbing miR-578 and elevating VEGF expressions [12]. Circ_0001658 expedites the viability and metastasis of OS cells via monitoring miR-382-5p/YB-1 axis [13].

Here, we adopted circRNA microarray to identify differentially expressed circRNAs in OS, and demonstrated that circ_0008259 was significantly down-regulated in OS. Here we prove that circ_0008259 is silenced in OS tissues and cells. Functionally, circ_0008259 restrains viability and metastatic potential of OS cells and promotes the apoptosis via modulating miR-21-5p / Programmed cell death 4 (PDCD4) pathway.

## RESULTS

### Circ_0008259 is down-regulated in OS

**Figure 1:**
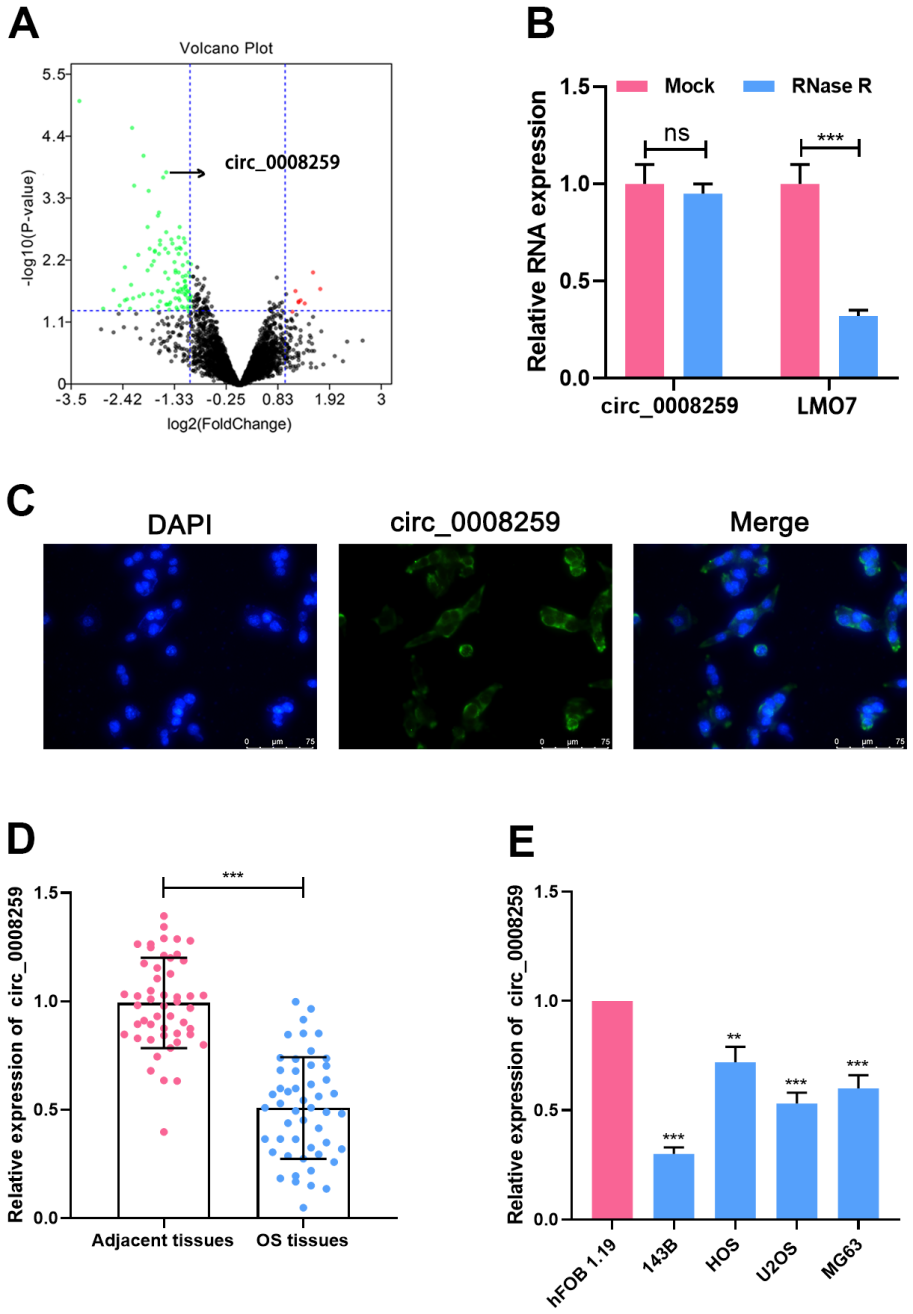
**Circ_0008259 expression level is downregulated in OS.** (**A**) The volcano plot showed that multiple circRNAs were up-regulated or down-regulated in OS tissues vs. non-cancerous tissues. Green plots represent down-regulated circRNAs, and red plots represent up-regulated circRNAs. (**B**) Circ_0008259 and linear LMO7 expression levels were detected by qRT-PCR after the total RNA was treated with RNase R. (**C**) Circ_0008259 was mainly presented in the cytoplasm of OS cells, which was verified by FISH. (**D**) qRT-PCR was used to detect the expression of circ_0008259 in OS tissues (n=50) and adjacent tissues (n=50). (**E**) qRT-PCR was used to detect the expression of circ_0008259 in normal osteoblast (hFOB1.19) and OS cell lines (143B, HOS, U2OS, and MG63). **P < 0.01, ***P < 0.001.

To identify differentially expressed circRNAs in OS, we analyzed a circRNA microarray (GSE96964) containing circRNA expression profiles of human OS cell lines (U2OS, U2OS/MTX300, HOS, MG63, 143B, ZOS, ZOSM) and human osteoblast hFOB1.19. With │log2FC│>1 and *P*<0.05 as the criteria, circRNAs with significantly abnormal expression in OS were obtained from 4660 circRNAs, of which 8 were raised and 102 were silenced (Figure 1A). Circ_0008259 (*P*=0.00016406, log_2_^FC^= -1.544423) was greatly depleted in the OS cell lines (Figure 1A). Circ_0008259 is derived from exons of the LIM domain 7 (LMO7) gene, and RNase R assay uncovered that circ_0008259 was much more stable than linear LMO7, suggesting circ_0008259 had a circular structure (Figure 1B). In addition, it was revealed that circ_0008259 was distributed in the cytoplasm of OS cells, revealing that it could probably be a competitive endogenous RNA (ceRNA) (Figure 1C). qRT-PCR uncovered that circ_0008259 expressions in 50 OS tissues were greatly lower than that in adjacent tissues (Figure 1D). In addition, as against normal osteoblast cells (hFOB1.19), circ_0008259 expressions in OS cell lines (143B, HOS, U2OS, and MG63) was greatly down-regulated (Figure 1E), being coherent to the findings of microarray analysis. Collectively, the abnormal silence of circ_0008259 was relevant to the progression of OS.

### Circ_0008259 depresses the malignant phenotypes of OS cells

**Figure 2:**
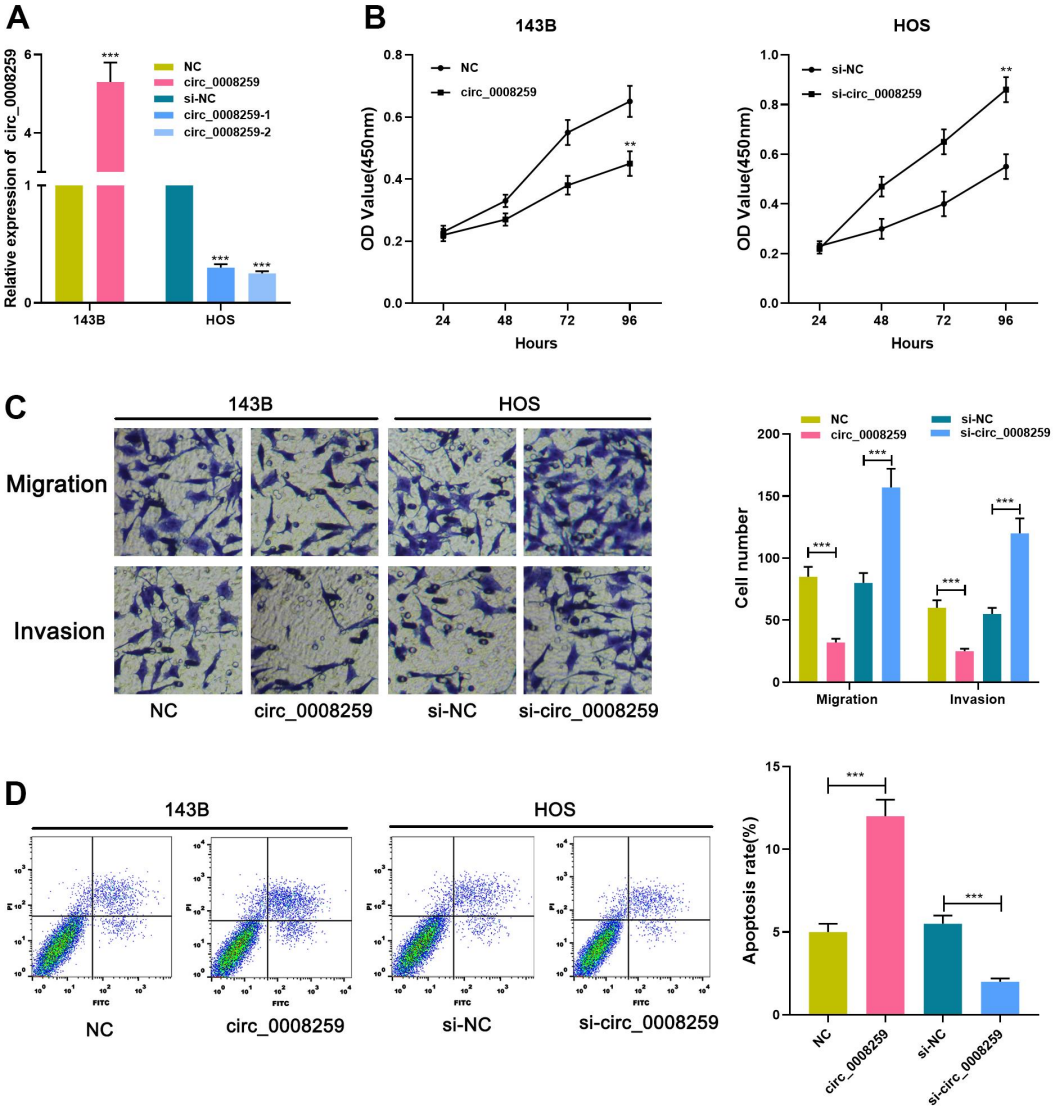
**Circ_0008259 inhibits the proliferation and metastatic potential of OS cells and promoted the apoptosis.** (**A**) qRT-PCR was employed to confirm that the circ_0008259 overexpression and circ_0008259 knockdown cell model was constructed successfully. (**B**) CCK-8 assay was used to detect the proliferation of OS cells (143B and HOS). (**C**) Transwell assay was used to detect the migration and invasion of OS cells (143B and HOS). (**D**) Flow cytometry was used to detect the apoptosis rate of OS cells (143B and HOS). **P < 0.01, and ***P < 0.001.

Circ_0008259 overexpressing plasmid was accordingly modeled and specifically transfected into 143B cell line, and si-circ_0008259-1/2 was subsequently transfected into HOS cell line, with the efficiency examined by qRT-PCR, and circ_0008259-1 with the best knockdown efficiency was adopted (Figure 2A). Additionally, CCK-8, transwell and flow cytometry highlighted that circ_0008259 overexpression depressed the malignant biological behaviors of 143B cells and promoted the apoptosis (Figure 2B–2D). Meanwhile, circ_0008259 silencing worked oppositely (Figure 2B–2D). Collectively, circ_0008259 served as a tumor deterrent in OS.

### Circ_0008259 negatively modulates miR-21-5p

**Figure 3:**
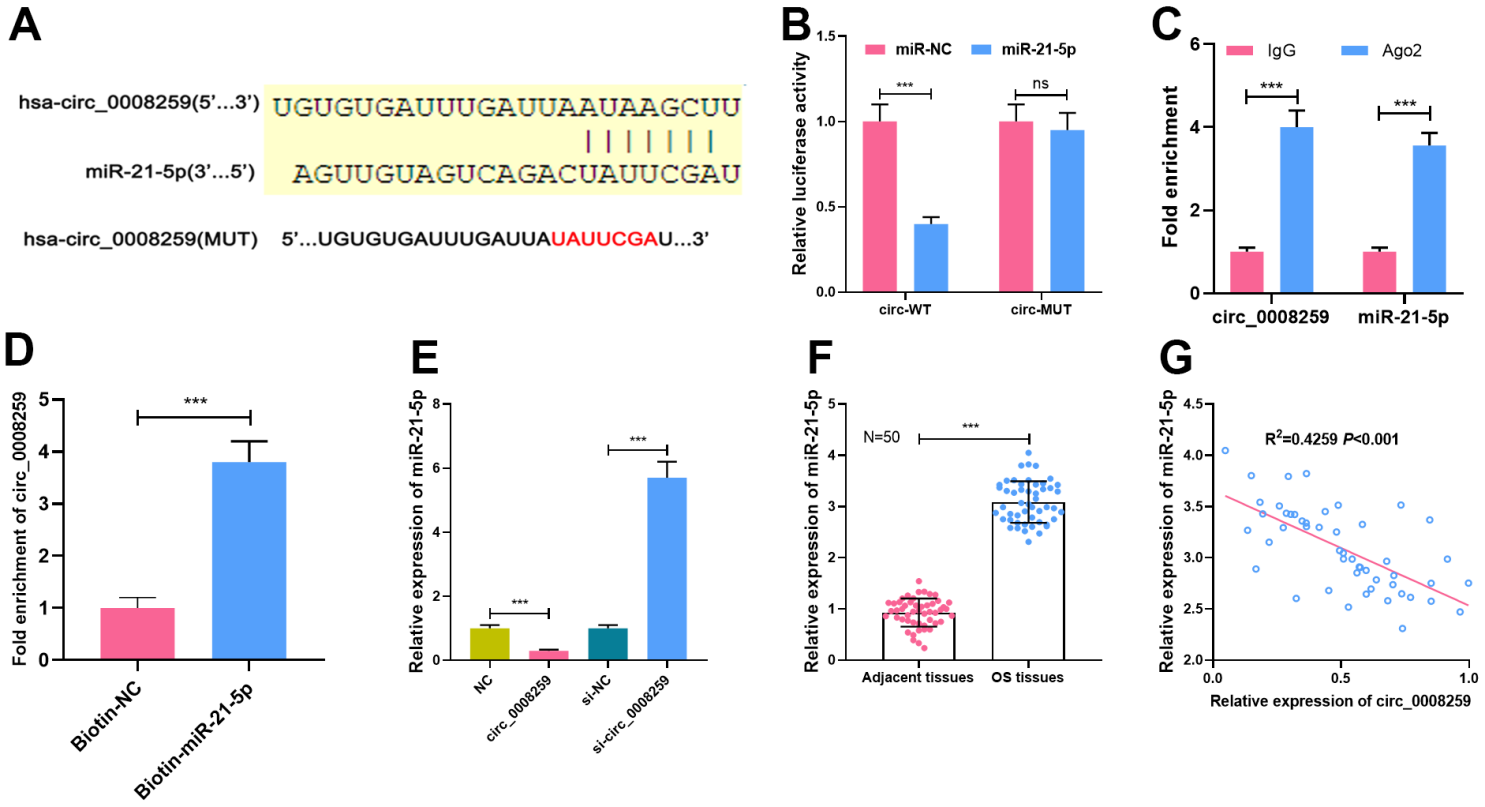
**MiR-21-5p is the target of circ_0008259.** (**A**) Circular RNA Interactome database was used to predict the binding site between circ_0008259 and miR-21-5p. (**B**) Dual-luciferase reporter assay showed that circ_0008259 adsorbed miR-21-5p. (**C**) Circ_0008259 and miR-21-5p co-immunoprecipitated with Ago2, which was revealed by RIP assay. (**D**) RNA pull-down assay showed that circ_0008259 interacted with biotin-labeled miR-21-5p. (**E**) qRT-PCR was used to detect the effects of overexpression or knockdown of circ_0008259 on miR-21-5p expression in 143B and HOS. (**F**) qRT-PCR was used to detect the expression of miR-21-5p in OS tissues (n=50) and adjacent tissues (n=50). (**G**) The expression of circ_0008259 was negatively correlated with the expression of miR-21-5p in OS tissues (R^2^=0.4259, P<0.001). ***P < 0.001.

Next, Circular RNA Interactome database showed that miR-21-5p was a potential target of circ_0008259 (Figure 3A). Besides, dual-luciferase reporter assay highlighted that miR-21-5p could restrain the luciferase activity of cells transfected with circ_0008259-WT, but that the of the cells transfected with circ_0008259-mut was not greatly impacted (Figure 3B). Besides, RIP assay uncovered that circ_0008259 and miR-21-5p were predominantly enriched in Ago2 group, as against IgG group (Figure 3C). RNA pull-down experiments showed that circ_0008259 could be silenced by biotin-labeled miR-21-5p, displaying that circ_0008259 can directly bind to miR-21-5p (Figure 3D). Besides, circ_0008259 overexpression repressed miR-21-5p expressions in OS cells, while circ_0008259 silencing shown a opposite effect (Figure 3E). Additionally, we observed that miR-21-5p expression in OS tissues was higher than that in adjacent tissues, which was negatively correlated with circ_0008259 expression (Figure 3F, 3G). This evidence highlighted that miR-21-5p was the target of circ_0008259 in OS cells.

### PDCD4 is a target of miR-21-5p

**Figure 4:**
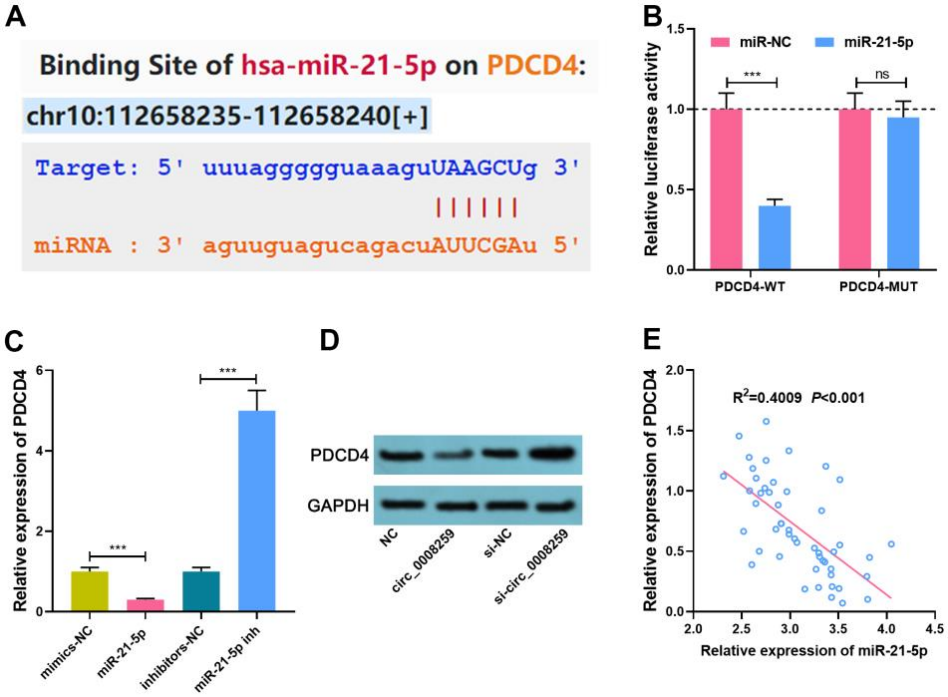
**MiR-21-5p specifically regulates PDCD4.** (**A**). StarBase database was used to predict the binding site between miR-21-5p and PDCD4. (**B**) Dual-luciferase reporter assay was used to confirm the interaction between miR-21-5p and PDCD4. (**C**, **D**) qRT-PCR and Western blot assays was used to detect the expression of PDCD4 after circ_0008259 and miR-21-5p were selectively regulated. (**E**) The expression of PDCD4 was negatively correlated with the expression of miR-21-5p in OS tissues (R^2^=0.4009, *P*<0.001). ***P < 0.001.

StarBase databases predicted that miR-21-5p could probably target PDCD4 3’UTR (Figure 4A). Besides, luciferase reporter assay depicted that miR-21-5p dramatically restrained the luciferase activity of PDCD4-WT reporter, while that of PDCD4-MUT reporter was less impacted (Figure 4B). qRT-PCR and western blot showed that miR-21-5p mimics demonstrably repressed PDCD4 mRNA and protein expression, while inhibition of miR-21-5p induced the elevation of PDCD4 in OS cells (Figure 4C, 4D). miR-21-5p was negatively pertinent to PDCD4 expressions in OS tissues (Figure 4E). Collectively, these studies suggested that PDCD4 was the target of miR-21-5p.

### Circ_0008259 represses OS progression via monitoring miR-21-5p / PDCD4 axis

**Figure 5:**
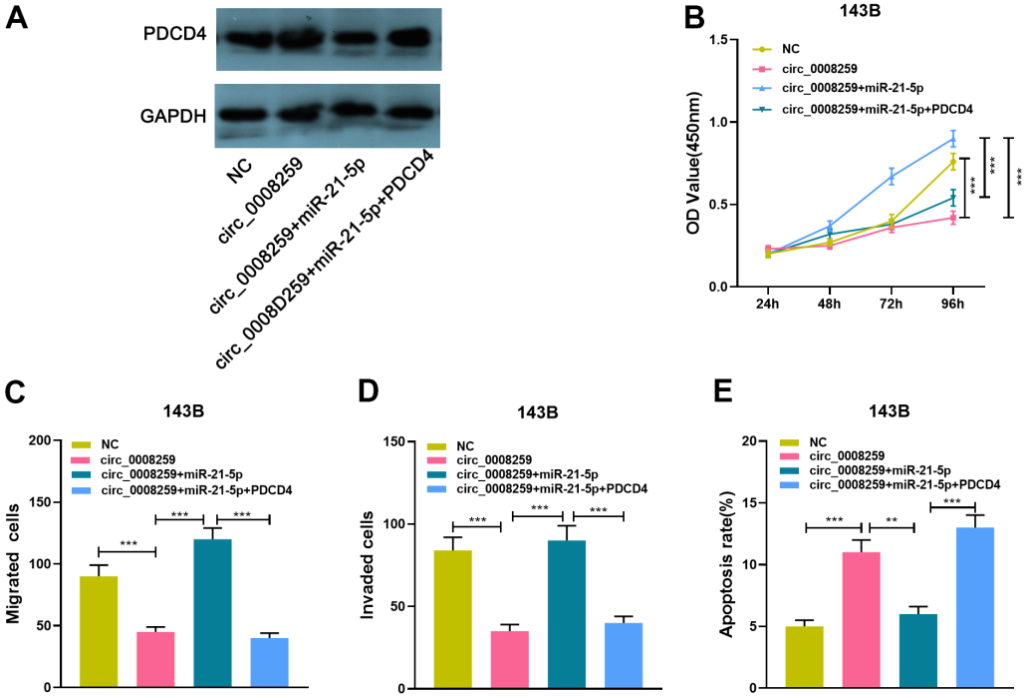
**Circ_0008259 represses OS progression via modulating miR-21-5p / PDCD4 axis.** (**A**) 143B cells with circ_0008259 overexpression was transfected with miR-21-5p mimics and PDCD4 overexpression vector, and the expression of PDCD4 in OS cells was detected by Western blot assay. (**B**) CCK8 assay was used to detect the proliferation of 143B cells after transfection. (**C**, **D**) Transwell assay was used to detect the migration and invasion of 143B cells after transfection. (**E**) Flow cytometry was used to detect the apoptosis rate of 143B cells after transfection. **P < 0.01, and ***P < 0.001.

**Figure 6:**
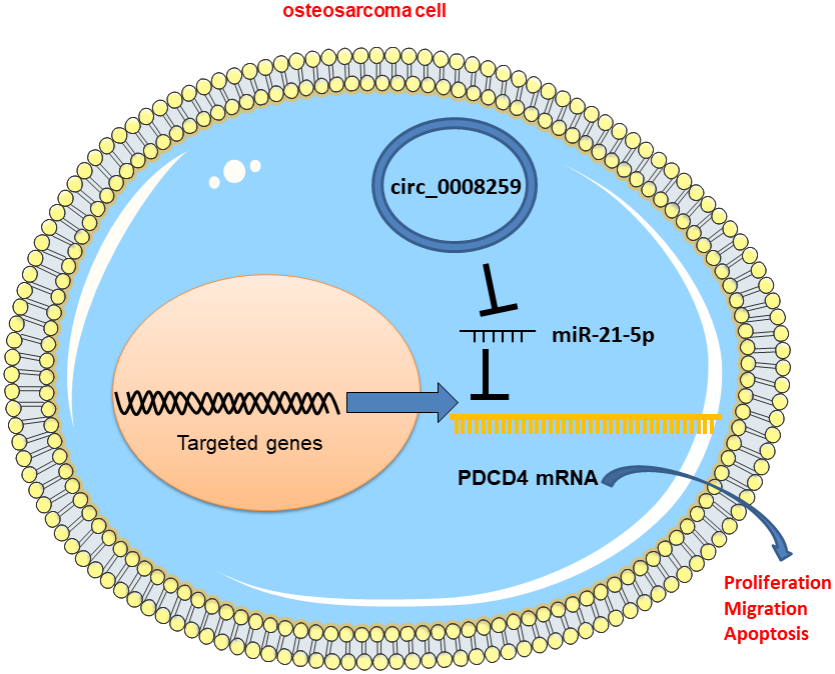
Graphic abstract: circ_0008259 functions as a ceRNA to regulate the expression of miR-21-5p and PDCD4 to block OS progression.

To expound the downstream mechanism of circ_0008259 in regulating the OS progression, we conducted a series of “rescue experiments” with 143B cells. Western blot showed that circ_0008259 overexpression could promote PDCD4 expression, which was weakened by the co-transfection of miR-21-5p mimics, while the co-transfection of PDCD4 overexpression plasmids enhanced PDCD4 protein expression (Figure 5A). As shown, circ_0008259 overexpression impeded the growth, migration and invasion and accelerated the apoptosis of 143B cells, while miR-21-5p mimics rescued this impact (Figure 5B–5E). Moreover, PDCD4 overexpression rescued the impacts of miR-21-5p restoration in 143B (Figure 5B–5E). In short, circ_0008259 suppressed the progression of OS via monitoring miR-21-5p / PDCD4 axis (Figure 6).

## DISCUSSION

CircRNAs, as reported, are not simply the junk-products in pre-mRNA splicing [14]. Recently, certain circRNAs, reportedly, are the key regulators in the physiological and pathological processes [15]. Unlike linear RNA, circRNAs are endowed with special covalently closed loop structures, which enables them more resistant to exonuclease-mediated degradation [6]. CircRNA can interfere with RNA binding protein (RBP) to monitor the functions of these proteins [16, 17]. CircRNA also partakes in the transcriptional or post-transcriptional gene regulation [18]. Importantly, some circRNA, as reported, are interfered in the post-transcriptional regulation of some genes via acting as "molecular sponge", thus reducing the ability of miRNA to bind with mRNA. CircRNAs, as reported, are abnormally expressed in OS, and feature prominently in the progression of OS [19, 20]. For example, circTADA2A is highly expressed in both OS tissue and cell lines, and facilitates the malignant biological behaviors of cancer cells through monitoring miR-203a-3p/CREB3 axis [21]. circ_ANKIB1 can activate the STAT3 pathway and the multiplication and invasion of OS cells via enhancing the regulation of miR-19b on the downstream target SOCS3 [22]. Here, we discovered that circ_0008259 was greatly depleted in OS. In addition, function experiments implied that circ_0008259 depressed the viability, migration and invasion of OS cells and expedites the apoptosis. This is the first project focusing on the role of circ_0008259 in OS. Interestingly, in a recent study, circ_0008259 is raised in gastric cancer tissues, and it promotes gastric cancer progression [23]. This suggests that in different cancers, the role of circ_0008259 is distinct.

MiRNAs are the key post-transcriptional regulators of gene expression, and miRNAs can regulate surpassing 50% protein-coding genes expression and be implicated in regulating almost all cellular processes [24]. Specific miRNA can serve as biomarkers of different human diseases including cardiovascular diseases, metabolic diseases, and tumors [25, 26]. For example, miR-206 expedites the multiplication and metastasis of OS cells via targeting Notch3 [27]. miR-624-5p can accelerate the tumorigenesis and advancement of OS by targeting PTPRB and repressing hippo signal transduction [28]. MiR-21-5p works as a cancer-promoter in diverse tumors [29–31]. For example, miR-21-5p facilitates the multiplication of non-small cell lung cancer cells via targeting TGFBI [29]. MiR-21-5p expedites the growth and invasion of colon adenocarcinoma cells by targeting CHL1 [30]. Importantly, miR-21-5p is elevated in OS and strengthens the viability and metastasis of OS cells [31]. Consistently, we proved that miR-21-5p was up-regulated in OS tissues and cell lines, and miR-21-5p was identified as the downstream target of circ_0008259, with its expressions inhibited by circ_0008259.

PDCD4, a tumor deterrent, is frequently down-regulated in diverse types of cancer [32]. As reported, PDCD4 is implicated in regulating gene translation and transcription. PDCD4, for example, may directly bind with c-Myb, Bcl-xL and XIAP mRNA to inhibit their translation, thus restraining cell multiplication and promoting the apoptosis [33]. In addition, PDCD4 interferes directly with the transcription factor Twist 1 and impedes cell growth via decreasing the Twist 1 target gene YB1 [34]. Additionally, it is reported that PDCD4 can inhibit the invasion of tumor cells via maintaining E-cadherin level [35]. PDCD4 expression is also negatively correlated with Ki-67 expression in giant cell tumors of the bone, suggesting that it may suppress tumor growth [36]. Some previous studies report that, miR-21-5p can specifically repress PDCD4 expression, thus promoting the progression of multiple cancers, including OS [31, 37, 38]. Here we observed that miR-21-5p could target and repress PDCD4; additionally, we demonstrated that, PDCD4 could be negatively regulated by circ_0008259. Our data support that the ceRNA network consisting of circ_0008259, miR-21-5p and PDCD4 is involved in OS progression.

To recapitulate briefly, circ_0008259 is under-expressed in OS. Functionally and mechanistically, circ_0008259 increases PDCD4 expression via adsorbing miR-21-5p, thus repressing the progression of OS. This work highlights that circ_0008259 be may a promising biomarker and therapeutic target for OS sufferers.

## MATERIALS AND METHODS

### Tissue samples

50 sufferers who were diagnosed as OS and underwent tumor resection in Shaanxi Provincial People’s Hospital were enrolled. OS samples and adjacent tissues were stored in liquid nitrogen. Specifically, this project was accordingly endorsed by the Ethics Committee of Shaanxi Provincial People’s Hospital.

### Expression profile analysis of circRNAs

GSE96964 dataset was from Gene Expression Omnibus database (GEO). Adjusted *P* < 0.05 and │log_2_^(Fold Change)^│>1 were wielded to differentiate the differentially expressed circRNAs.

### Cell culture and cell transfection

Human osteoblast cells (hFOB1.19) and OS cell lines (143B, HOS, U2OS, and MG63) available from the American Type Culture Collection (Rockville, MD, USA) were followingly cultured in Dulbecco’s modified Eagle’s medium (Invitrogen, Carlsbad, CA, USA) with 10% fetal bovine serum at 37° C in 5% CO_2_. The sequence of circ_0008259 or PDCD4 was cloned by polymerase chain reaction (PCR) and inserted into the pcDNA3.1 vector to construct the circ_0008259 or PDCD4 overexpression plasmid. The small interfering RNA (siRNA) targeting circ_0008259 (si-circ_0008259-1/2), miR-21-5p mimic and inhibitor, and the negative controls (empty vector, si-NC, or miR-control) were from GenePharma (Shanghai, China). OS cells were transiently transfected by Lipofectamine 2000 (Invitrogen).

### Quantitative real-time polymerase chain reaction (qRT-PCR)

Total RNA extracted by TRIzol reagent (Vazyme, Nanjing, China) were reversely transcribed to complementary DNA (cDNA) with a PrimeScript™ RT Master Mix kit (Takara, Otsu, Japan). Circ_0008259 and PDCD4 expressions were determined with a SYBR Green qRT-PCR kit (Takara), and miR-21-5p expressions were determined with the stem-loop primer SYBR Green qRT-PCR kit (Synbio Tech, Suzhou, China). Glyceraldehyde 3-phosphate dehydrogenase (GAPDH) and U6 were used as internal controls for circRNA/mRNA and miRNA, respectively. Subsequently, qRT-PCR was performed on an ABI 7500 Fast Real-Time PCR system (Applied Biosystems, Foster City, California, USA), with the relative expression estimated by 2^−ΔΔCT^. Primer sequences are in Table 1. To determine the circular structure of circ_0008259, total RNA (2 μg) was generally incubated for 30 min at 37° C with 3 U/μg of RNase R (Epicentre Technologies, Madison, WI, USA). Cells were specifically treated with RNase R, and qRT-PCR was accomplished to detect circ_0008259 expressions.

**Table 1:** Sequences used for qRT-PCR.

**Name**	**Primer sequences**
circ_0008259	Forward: 5'-AAGAAGCCCAGCTTTTCCAT-3'
	Reverse: 5'-TCACACAGCAGAACACCATTT-3'
miR-21-5p	Forward: 5'-GTGCAGGGTCCGAGGT-3'
	Reverse: 5'-GCCGCTAGCTTATCAGACTGATGT-3'
PDCD4	Forward: 5'-CGACAGTGGGAGTGACGCCCTTA-3'
	Reverse: 5'-CAGACACCTTTGCCTCCTGCACC-3'
LMO7	Forward: 5'-AATCAGCATAAACCAGACGCC-3'
	Reverse: 5'-CTGGGCTACCTGCTTCAACT-3'
U6	Forward: 5'-TGCGGGTGCTCGCTTCGGCAGC-3'
	Reverse: 5'-CCAGTGCAGGGTCCGAGGT-3'
GAPDH	Forward: 5'-GCACCGTCAAGGCTGAGAAC-3'
	Reverse: 5'-TGGTGAAGACGCCAGTGGA-3'

### Fluorescence *in situ* hybridization (FISH)

Briefly, OS cells were accordingly fixed in 4% paraformaldehyde for 10 min and followingly rinsed with phosphate-buffered saline (PBS). Besides, cells were generally permeabilized with 0.5% Triton X-100 for 15 min at 4° C. What’s more, digoxigenin (DIG)-labeled circ_0008259 probe or control probe mixture was then incubated with OS cells for 4 h at 37° C. Additionally, the nuclei were specifically stained with 4,6-diamidino-2-phenylindole (DAPI) staining solution for 30 min at ambient temperature. Ultimately, cells were generally observed with a confocal laser scanning microscope.

### Cell counting Kit-8 (CCK-8) assay

Transfected cells were inoculated into 96-well plates (1×10^3^ / well) and subsequently cultured. At 24 h, 48 h, 72 h and 96 h, cells were mixed with 10 μL of CCK-8 solution (Dojindo, Kumamoto, Japan) and followingly incubated for 2 h at ambient temperature. The value of OD_450nm_ was registered by a microplate reader (Bio-Rad, Hercules, CA, USA).

### Transwell assay

As to migration assay, transfected cells re-suspended in serum-free DMEM were inoculated into Transwell chamber (Corning Costar, Cambridge, MA, USA) (1×10^5^ cells/well), and the lower part was loaded with 250 μl of medium containing 10% fetal bovine serum. 48 h later, the chamber was removed out. Notably, cells on the upper were scrapped with cotton swabs, and those on the bottom were followingly fixed with 4% paraformaldehyde, and accordingly stained with crystal violet solution for 15 min. Then cell was accordingly immersed in PBS, dried, and observed under a microscope. As to invasion assays, the filter was generally pre-coated with a layer of diluted Matrigel, and the rest processes were executed as described above.

### Flow cytometric analysis

The apoptosis was subsequently detected with an Annexin V-FITC Apoptosis Detection kit (Invitrogen). Transfected cells were accordingly immersed in PBS, then re-suspended in 200 μL of binding buffer (1×10^6^ cells/ml). Besides, cells in each sample were generally stained with 10 μL of Annexin V-FITC staining solution and 5 μL of propidium iodide (PI) staining solution at ambient temperature for 20 min in darkness. Next, the cell apoptosis was probed by a flow cytometer (BD Biosciences, San Jose, CA, USA).

### Luciferase reporter gene assay

Circ_0008259 and PDCD4 3’UTR sequences containing wild type (WT) and mutant (MUT) miR-21-5p binding sites were subsequently synthesized and followingly inserted into pGL3 luciferase vectors (Promega, Madison, WI, USA) to create the luciferase reporter vectors. Circ_0008259-WT, circ_0008259-MUT, PDCD4-WT or PDCD4-MUT was accordingly co-transfected with miR-21-5p mimic or control miRNA into HEK293T cells, respectively. 48 h later, the firefly and Renilla luciferase activities were probed by dual-luciferase reporter gene assay (Promega).

### RNA-binding protein immunoprecipitation (RIP) assay

RIP assay was conducted by the EZ-Magna RIP Kit (Millipore, Billerica, MA, USA) as instructions. RIP lysis buffer with proteinase and RNase inhibitors were adopted to lyse the OS cells. Next, the lysates were followingly incubated with magnetic beads conjugated with human anti-Ago2 antibody or control anti-immunoglobulin G (IgG) antibody. Next, the immunoprecipitate was obtained, and proteinase K was used to remove proteins. Next, qRT-PCR was wielded to probe circ_0008259 and miR-21-5p expressions.

### RNA pull-down assay

Cells were followingly transfected with 50 nM biotinylated WT-bio-miR-21-5p or MUT-bio-miR-21-5p. 48 h later, cells were subsequently harvested and immersed in PBS. Notably, cells were accordingly lysed in lysis buffer (Ambion, Austin, Texas, USA), and the lysate was generally incubated with M-280 streptavidin magnetic beads pre-coated with RNase-free BSA and yeast tRNA. After incubation at 4° C for 3 h, the beads were specifically rinsed twice with pre-cooled lysis buffer, thrice with low salt buffer, and once with high salt buffer. Besides, the bound RNA was subsequently purified by TRIzol method, with circ_0008259 expression subjected to RT-qPCR.

### Western blotting analysis

The total protein of transfected cells were obtained by radioimmunoprecipitation lysis buffer (Beyotime, Shanghai, China), and then the protein samples were subsequently separated by sodium dodecyl sulfate-polyacrylamide gel electrophoresis, and then the proteins were accordingly transferred onto a polyvinylidene fluoride membrane (Millipore), which were then blocked with 5% skimmed milk for 1 h at ambient temperature. Notably, the membranes were specifically incubated with rabbit anti-PDCD4 antibody (1:1000, ab51495, Abcam, Cambridge, UK) or mouse anti-GAPDH antibody (1:2000, ab8245, Abcam, Cambridge, UK) at 4° C overnight, and then with HRP-conjugated secondary antibodies at ambient temperature for 1 h. Ultimately, the protein bands were developed by an enhanced chemiluminescence kit (Pierce, Waltham, MA, USA).

### Statistical analysis

Graphs were accordingly generated by GraphPad Prism 8.0 (GraphPad Software, La Jolla, CA, USA), and analysis was followingly fulfilled by SPSS 22.0 (IBM, Chicago, IL, USA). All trials were in triplicate, and the data were displayed as the mean ± standard deviation. Student’s *t*-test or one-way ANOVA was adopted for making comparisons. Besides, spearman’s correlation coefficient was using to evaluate the correlations among circ_0008259, miR-21-5p and PDCD4 in OS tissues. Statistically, *P* <0.05 was meaningful.

### Ethics statement

Our study was approved by the Ethics Review Board of Shaanxi Provincial People’s Hospital.

### Data availability statement

The data used to support the findings of this study are available from the corresponding author upon request.
